# Injectable Methacrylated Gelatin Hydrogel for Safe Sodium Hypochlorite Delivery in Endodontics

**DOI:** 10.3390/gels9110897

**Published:** 2023-11-13

**Authors:** Renan Dal-Fabbro, Yu-Chi Huang, Priscila T. A. Toledo, Leticia C. Capalbo, Rhima M. Coleman, Hajime Sasaki, J. Christopher Fenno, Marco C. Bottino

**Affiliations:** 1 Department of Cariology, Restorative Sciences and Endodontics, School of Dentistry, University of Michigan, Ann Arbor, MI 48109, USA; renandf@umich.edu (R.D.-F.); yuchicat@umich.edu (Y.-C.H.); priscilt@umich.edu (P.T.A.T.); lcapalbo@umich.edu (L.C.C.); hajimes@umich.edu (H.S.); 2 Department of Preventive and Restorative Dentistry, School of Dentistry, São Paulo State University (UNESP), Aracatuba 16015-050, SP, Brazil; 3 Department of Biomedical Engineering, College of Engineering, University of Michigan, Ann Arbor, MI 48109, USA; rhimacol@umich.edu; 4 Department of Mechanical Engineering, University of Michigan, Ann Arbor, MI 48109, USA; 5 Department of Biologic and Materials Sciences & Prosthodontics, School of Dentistry, University of Michigan, Ann Arbor, MI 48109, USA; fenno@umich.edu

**Keywords:** regenerative endodontics, sodium hypochlorite, infection, hydrogel, drug delivery, dentistry

## Abstract

Keeping sodium hypochlorite (NaOCl) within the root canal is challenging in regenerative endodontics. In this study, we developed a drug delivery system using a gelatin methacryloyl (GelMA) hydrogel incorporated with aluminosilicate clay nanotubes (HNTs) loaded with NaOCl. Pure GelMA, pure HNTs, and NaOCl-loaded HNTs carrying varying concentrations were assessed for chemo-mechanical properties, degradability, swelling capacity, cytocompatibility, antimicrobial and antibiofilm activities, and in vivo for inflammatory response and degradation. SEM images revealed consistent pore sizes of 70–80 µm for all samples, irrespective of the HNT and NaOCl concentration, while HNT-loaded hydrogels exhibited rougher surfaces. The hydrogel’s compressive modulus remained between 100 and 200 kPa, with no significant variations. All hydrogels demonstrated a 6–7-fold mass increase and complete degradation by the seventh day. Despite an initial decrease in cell viability, all groups recovered to 65–80% compared to the control. Regarding antibacterial and antibiofilm properties, 12.5 HNT(Double) showed the highest inhibition zone on agar plates and the most significant reduction in biofilm compared to other groups. In vivo, the 12.5 HNT(Double) group displayed partial degradation after 21 days, with mild localized inflammatory responses but no tissue necrosis. In conclusion, the HNT-NaOCl-loaded GelMA hydrogel retains the disinfectant properties, providing a safer option for endodontic procedures without harmful potential.

## 1. Introduction

Cavities are among the most prevalent worldwide conditions, with ninety percent of adults over twenty having had at least one event [1]. If not properly initially treated, they can compromise the tooth’s inner structure (pulp tissue), later requiring a root canal (endodontic) treatment to prevent the loss of the whole element. The chemo-mechanical approach of endodontic therapy involves cleaning and disinfecting the inner tooth space, removing the infected or inflamed pulp, filling, and sealing it. However, the difficulty of this procedure can vary depending on the tooth eruption stage since a permanent immature tooth can take approximately three years after the eruption to complete root development and apical closure [2]. Recently, endodontic treatment of immature teeth has shifted towards a strategy that aims to regenerate the damaged and necrotic tissues with newly formed, healthy, and functional intracanal tissue. This approach focuses on creating regenerated tissue similar to the dental pulp, i.e., being vascularized, innervated, and capable of generating new odontoblasts, resulting in new dentin matrices that can be mineralized [3,4]. Although several materials have been developed to promote hemostasis [5], healing [5], and bone regeneration [6], it is crucial to achieve successful root canal disinfection since a previous infection may damage tissue-forming cells and periapical stem cells, negatively influencing pulp regeneration and leading to tissue formation of periodontal origin rather than pulp–dentin complexes [7,8].

The latest guidelines for treating immature teeth with endodontic procedures suggest minimal mechanical instrumentation due to the short and thin dentin walls preventing further weakening and fracture [2,9]. Instead, chemical approaches such as abundant irrigation procedures and internal dressing materials are recommended for proper disinfection [9]. Due to its antimicrobial properties and capacity to dissolve organic matter, sodium hypochlorite (NaOCl) is widely used as an endodontics irrigant [10]. Additionally, it is inexpensive, readily available, and convenient to store, making it a popular irrigant [11]. However, several factors must be considered in young patients with immature teeth. Firstly, the diminutive root length, inverse canal taper, large pulp chambers, and open apex with lack of apical stop can result in accidental extrusion and absorption, leading to severe complications such as pain, soft tissue irritation, and necrosis [12,13]. Secondly, the pulp tissue in young teeth is highly vascular and innervated, rendering it more sensitive to the cytotoxic effects of NaOCl, causing irreversible damage to the vital pulp that can affect root development continuity and dentinogenesis. Additionally, children can be more challenging to manage and less cooperative, especially when long treatment periods are required [12]. Hence, while NaOCl is an effective endodontic irrigant, its use in young teeth necessitates careful consideration of dosage, application techniques, and precautions to minimize the risks and ensure optimal outcomes [8].

Gelatin methacryloyl (GelMA) hydrogels have emerged as a promising platform for drug delivery in recent years, providing biocompatibility (ability to promote cell adhesion via arginine–glycine–aspartic acid, RGD sequences), biodegradability (in response to matrix metalloproteinases, MMPs), mechanical stability, and controlled drug release properties [14]. Tailoring the swelling and degradation behaviors of GelMA hydrogels allows for customizing specific therapeutic requirements, highlighting their tunable properties. In addition to GelMA, aluminosilicate clay nanotubes (halloysite, HNTs) have emerged as a promising strategy to enhance drug loading in several drug delivery systems [15]. HNTs are cylindrical nanostructures possessing a unique structure, allowing them to act as nanocarriers with a high aspect ratio and a hollow core, providing a large surface area for drug adsorption [16].

Considering the aspects mentioned above of both materials, there is a significant interest in developing a delivery system that can facilitate controlled and efficient administration of antimicrobial doses of NaOCl for regenerative endodontic treatment in permanent immature teeth. Therefore, this study aimed to devise an injectable and biodegradable GelMA hydrogel incorporated with NaOCl-loaded HNTs at various concentrations. This novel approach aims to enable the continuous and safe delivery of NaOCl for endodontic therapies, ensuring effective disinfection while minimizing the risks of injuries and cytotoxicity.

## 2. Results and Discussion

### 2.1. Chemo-Morphological Characterization

**Figure 1:**
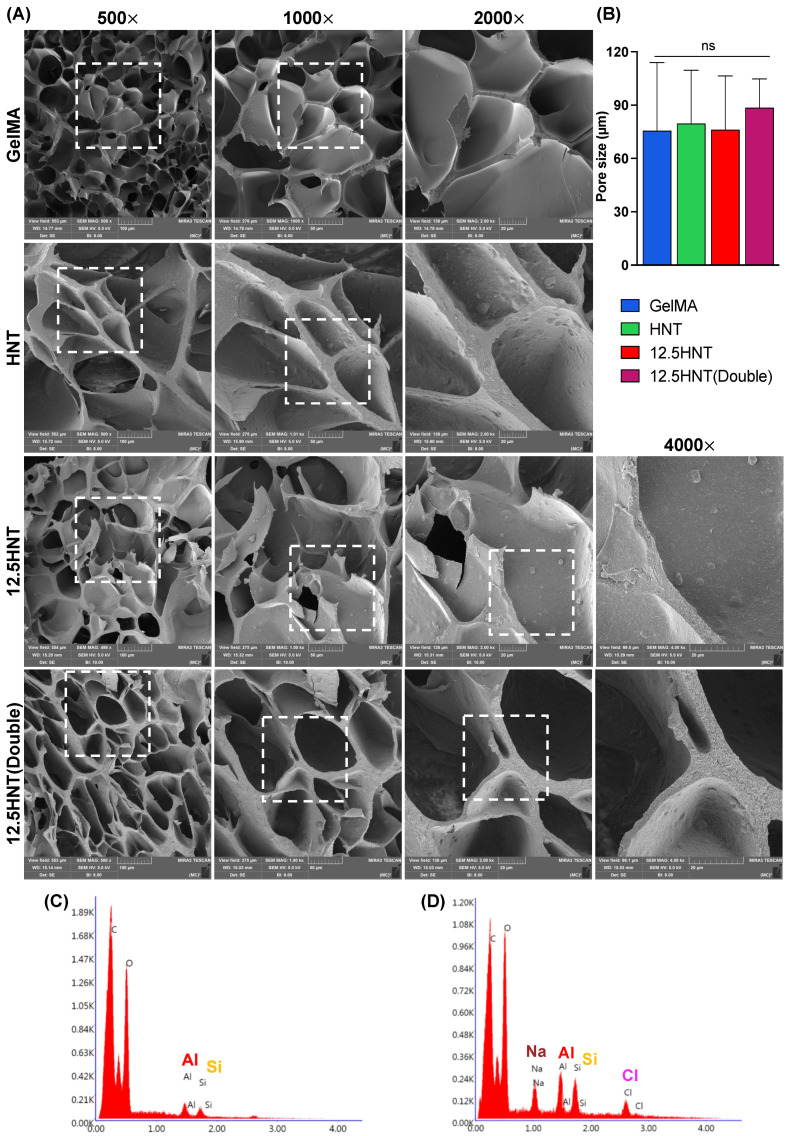
Morphological and chemical analyses of the nanotube-modified GelMA-based hydrogels. (**A**) SEM hydrogel cross-sections from GelMA, GelMA modified with nanotubes (HNT group), GelMA modified with 12.5% NaOCl-loaded nanotubes at 5% (*w*/*v*) and Double (10% *w*/*v*). The 4000× magnification shows the uniform presence of nanotubes on the hydrogel surface. (**B**) Mean pore size and standard deviation in µm—one-way ANOVA//Tukey’s post-hoc, α = 5%, ns: not significant. (**C**,**D**) EDS results confirm the presence of Si and Al characteristics from the aluminosilicate nanotubes in the HNT group and the additional presence of Na and Cl, attesting to the successful sodium hypochlorite incorporation in the 12.5 HNT group.

The mechanical properties of endodontic materials play a crucial role in their effectiveness and long-term stability. To characterize the hydrogel, various parameters were examined, including its morphological features through cross-section scanning electron microscopy (SEM) micrographs. It is important to note that freeze–drying can influence the material’s porosity; therefore, consistent treatment was applied to all samples to maintain uniformity [15]. The SEM micrograph in Figure 1A reveals that pure GelMA has a highly porous structure with an average pore size of approximately 70 to 80 µm. When HNTs were incorporated into GelMA, the pore size remained unchanged, but a rougher surface than of pure GelMA was noted since HNTs can be evenly distributed throughout the hydrogel structure without affecting its overall porous dimension. In addition, NaOCl-loaded HNTs also had no impact on the pore size. The pore size of a hydrogel is essential because it can affect the physical, chemical, and biological properties of the hydrogel. It also can affect the rate and extent of diffusion of molecules through the hydrogel and the ability of cells to grow and migrate through it. If the pores are too small, it may limit the diffusion of larger molecules, while larger pores may allow for the diffusion of larger molecules. Mechanically, the pore size plays an important role in interfering with stiffness and elasticity since it can affect the crosslinking density, the network structure, and, consequently, the swelling behavior of the hydrogel [16]. To confirm the presence of NaOCl-loaded nanotubes, energy-dispersive spectroscopy (EDS) was performed. The components of NaOCl and HNTs (Aluminum and Silicate) are clearly labeled in Figure 1C,D for the HNT and 12.5 HNT groups, respectively.

### 2.2. Mechanical Characterization

**Figure 2:**
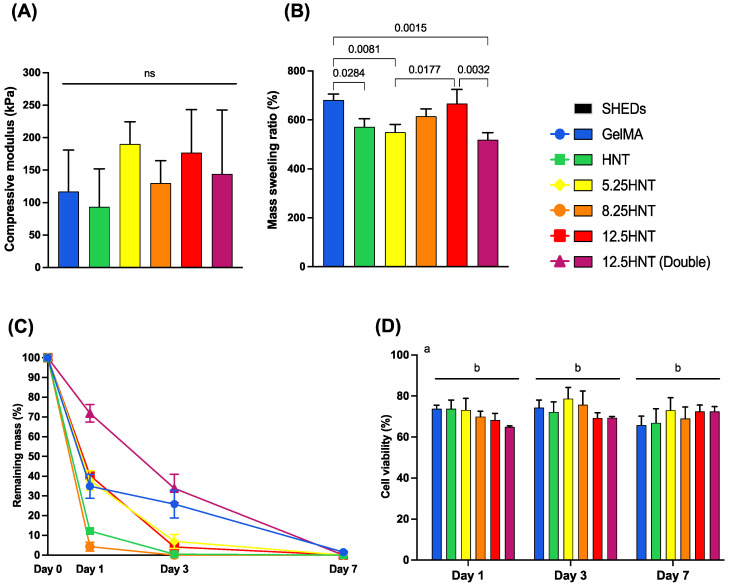
Biomechanical properties of GelMA-based hydrogels. (**A**) Compressive modulus (in kPa—*n* = 5). (**B**) The mass swelling ratio of GelMA and GelMA modified with nanotubes loaded with NaOCl after incubation in DPBS at 37 °C (*n* = 4). (**C**) The in vitro biodegradation of GelMA and GelMA-modified groups incubated in DPBS containing 1 U/mL of collagenase type I at 37 °C over time (*n* = 4). (**D**) Cell viability (%) of SHEDs in response to aliquots from day 1 to day 7 from NaOCl-loaded nanotube-modified GelMA-based hydrogels (*n* = 5). All the results are displayed as mean ± SD. Different letters denote significant differences between compared groups. One-way ANOVA//Tukey’s post-hoc (**A**,**B**), and two-way ANOVA/Tukey’s post-hoc (**D**), α = 5%, ns: not significant.

The evaluation of the compressive modulus of hydrogels is crucial for medical applications. It determines the hydrogel’s mechanical stability and ability to withstand deformation under applied forces. The compressive modulus helps ensure that hydrogels used in tissue engineering or load-bearing scenarios mimic the mechanical properties of natural tissues and provide the necessary support. It is also crucial for controlled drug delivery, affecting drug release kinetics, system performance, and cell function and differentiation [15,17]. In the present investigation, to determine the effect of the concentration of NaOCl on the mechanical properties of the GelMA hydrogels, unconfined compression was performed on samples with different concentrations of NaOCl (Figure 2A). The hydrogel’s compressive modulus was consistently between 100 kPa and 200 kPa across all groups, with no significant differences observed. This indicates that the material’s mechanical properties did not change when HNTs or NaOCl-loaded HNTs were added to the GelMA framework, compared to the pure GelMA.

### 2.3. Swelling and Enzymatic Degradation

The swelling characteristics of a hydrogel play a vital role in various applications, influencing solute diffusion, surface properties, mechanical properties, and surface mobility [18]. Additionally, the swelling ratio provides valuable insights into the network structure of hydrogels since a higher one typically indicates a network structure with larger pores and greater interstitial volume, which can make the hydrogel more susceptible to degradation due to the formation of fewer cross-links [19]. Generally, all the groups experienced a mass increase of approximately six to seven times their original weight. The swelling ratio indicated that adding pure HNTs, 5.25 HNTs, and 12.5 HNTs(Double) to GelMA significantly decreased swelling compared to pure GelMA. Moreover, there was a tendency for an increase from the 5.25 HNT to 12.5 HNT group, but the 12.5 HNT(Double) group disrupted this. However, no significant differences were found between pure GelMA and the 8.25 HNT and 12.5 HNT groups (Figure 2B). The proposed hydrogel is specifically designed for application inside the root canal. The results are promising and alleviate concerns as they outperformed conventional pasty dressings. Moreover, the hydrogel will benefit from the protection the root dentin wall provides, shielding it from masticatory forces. This reinforces its potential as a suitable and effective solution for root canal treatments.

Understanding the hydrogel degradation behavior helps select suitable formulations, maintain structural integrity, optimize drug release kinetics, and identify potential risks or adverse effects for safe dental applications. In this study, the degradation profiles of GelMA and the effect of NaOCl-loaded nanotube incorporation were determined using collagenase type I, as previously reported [15,20]. The degradation pattern of the tested hydrogels over seven days is presented in Figure 2C. The 12.5 HNT(Double) group showed lower degradation rates than the other groups after 1 and 3 days. This can be attributed to the high amount of the nanotubes loaded in this hydrogel (10% *w*/*v*) compared to the other groups. Apart from this group, all the other HNT-loaded groups degraded faster than the pure GelMA, with no apparent pattern between the different NaOCl concentrations. By the third day, most of the hydrogels modified with NaOCl-loaded nanotubes had lost over 90% of their mass. All the hydrogels developed were wholly degraded by the seventh day. This outcome is satisfactory as the purpose of the hydrogel was to decrease bacterial contamination in the canal by safely releasing NaOCl. Typically, a follow-up appointment is scheduled after seven days to eliminate any remaining medication or hydrogel and continue with the subsequent stages of endodontic treatment [8].

### 2.4. Cytocompatibility

To validate the usage of the hydrogel system as an on-demand drug delivery system, it is crucial to determine the in vitro cytotoxicity. SHEDs were used in this experiment to assess cell viability. The cell viability for different groups at all time points had a significant statistical difference from the control group (SHEDs cultured on tissue culture plates), with no significant difference between the groups. Nonetheless, the overall data showed a cell viability around 65−80% for all groups at all time points tested (Figure 2D). Specifically, after one day the more highly concentrated 12.5 HNT(Double) group showed 64.96 ± 1.37% viability compared to control. However, the viability rose to cytocompatible levels from aliquots collected on day 3 and onwards (>70%). Usually, in the root canal procedure, the highest recommended concentration of NaOCl is around 5.25% for conventional root canal treatments and 1.5–3% for regenerative endodontic approaches [9]. However, in this project, NaOCl was loaded into the nanotubes, which affected the concentration and the speed at which it was released. In this way, despite the minor reduction from the 70% mark for aliquots collected on day 1, the hydrogels allowed the cells to recover and surpass the standard threshold, showing a cytocompatibility profile. The hydrogel system with high concentrations of NaOCl did not harm the cells as much as conventional NaOCl solutions since a recent study showed that using a 0.05% NaOCl solution for only two hours reduced SHED cell viability by 75% [21].

### 2.5. Antibacterial Properties

The main goal of endodontic treatment is to eradicate bacteria, virulence factors, and toxins, facilitating the resolution of inflammation and reducing inflammatory cell activity within the region [15]. Therefore, appropriate antimicrobial action is one of the most critical properties in this project since it can demonstrate whether the material is functional. This study focused on *E. faecalis*, a facultative anaerobic bacterium commonly found in pathologically affected root canals. This species is known for its high resistance to various antimicrobial agents, making it a challenging pathogen and a potential cause of root canal treatment failure [22,23]. The agar diffusion test is a highly recognized and extensively employed method for assessing the antimicrobial efficacy of endodontic irrigants, with a particular focus on their effectiveness against *E. faecalis*. This bacterium has been widely utilized in previous studies as a reliable indicator for evaluating the performance of endodontic irrigants, mainly due to its remarkable resistance to a broad spectrum of antimicrobial agents. This resilience underscores the significance of *E. faecalis* in accurately gauging treatment efficacy and formulating effective antimicrobial strategies in the context of root canal therapy [22].

**Figure 3:**
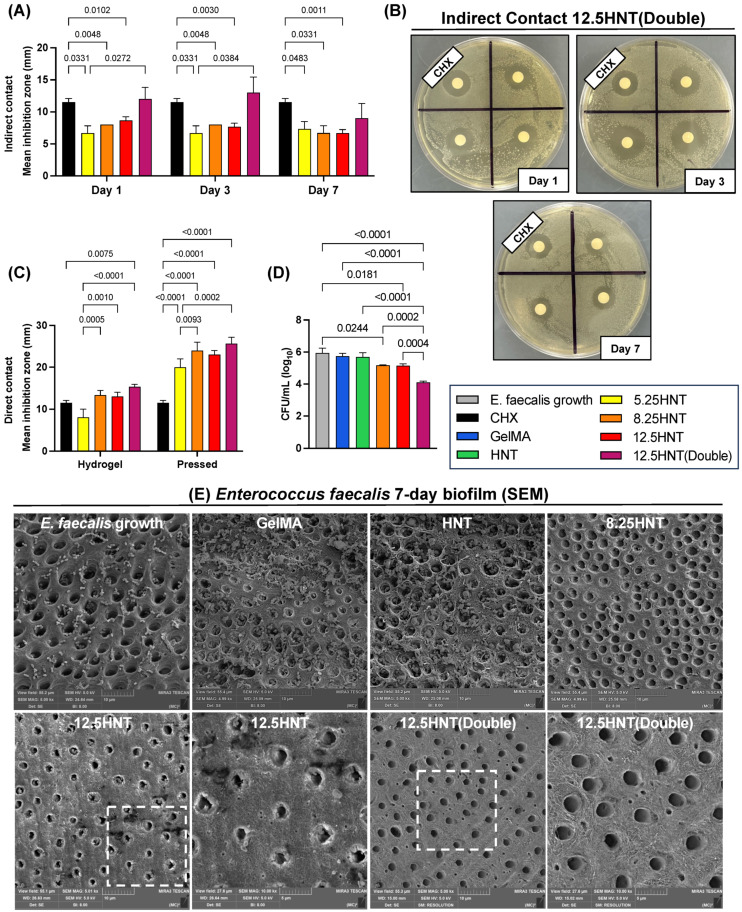
Antibacterial and antibiofilm evaluation. (**A**) Indirect contact of NaOCl-loaded nanotube-modified GelMA hydrogel through aliquots obtained after incubation in PBS with the on-demand challenge (1 U/mL collagenase type I) over 7 days. (**B**) Agar plates displaying the inhibition zone for the 12.5 HNT(Double) group over three time points. (**C**) Direct contact of NaOCl-loaded nanotube-modified GelMA hydrogel and pressed HNT-loaded powder. Results from the agar diffusion assays are represented as mean inhibition zone (in mm) against *E. faecalis*. Chlorhexidine (CHX) was used as a positive control. (**D**) Colony-forming units (CFU) of *E. faecalis* after hydrogel application. The 8.25 HNT, 12.5 HNT, and 12.5 HNT(Double) NaOCl-loaded GelMA-based hydrogels significantly reduced bacterial numbers compared to the untreated group. Two-way ANOVA/Tukey’s post-hoc (**A**–**C**) and one-way ANOVA//Tukey’s post-hoc (**D**), α = 5%. (**E**) SEM micrographs of bacterial biofilm (*E. faecalis* growth) on dentin surfaces: untreated dentin, dentin treated with the groups pristine GelMA, HNT, 8.25 HNT, 12.5 HNT, and 12.5 HNT(Double).

To test the hypothesis regarding the functionality of the proposed hydrogel as an on-demand drug delivery system, agar diffusion assays were conducted to assess the effects of direct contact (pressed disk and hydrogel) and indirect contact (aliquots) with NaOCl-loaded nanotube-modified hydrogels on *E. faecalis* [24]. In indirect contact, higher concentrations of NaOCl (12.5 HNT(Double) group) performed significantly better than lower concentrations on Day 1 and Day 3, showing similar values as chlorhexidine (Figure 3A,B). Notably, after seven days of incubation, the aliquots collected produced different results, probably due to a low concentration remaining. The 5.25 HNT, 8.25 HNT, and 12.5 HNT groups showed lower indirect antibacterial potential than chlorhexidine at all time points. As expected, direct contact results showed a significant bacterial reduction depicted by the larger inhibition zones than indirect contact, possibly due to the NaOCl content not being diluted in the PBS sample. In this analysis, we noted a marked increase in the inhibition zone for the higher concentrations; however, only the 12.5 HNT(Double) group was statistically higher than chlorhexidine in hydrogel or pressed forms (Figure 3C).

### 2.6. Antibiofilm Properties

When evaluating the effectiveness of hydrogels in combatting root canal infections, it is crucial to utilize a biofilm model. This is because biofilms possess a much greater resilience to antimicrobial agents and host defense mechanisms than their planktonic counterparts. Therefore, assessing the antimicrobial properties of hydrogels using a biofilm model provides a more accurate understanding of its potential efficacy in treating such infections [25,26]. For our research, we utilized a 7-day-old *E. faecalis* biofilm due to its significant role in causing endodontic diseases and its ability to form robust biofilms [20,27]. As shown in Figure 3D,E, it was unsurprising that no biofilm reduction was observed in the samples treated with pure GelMA and pure HNT groups.

The samples treated with the 8.25 HNT, 12.5 HNT, and 12.5 HNT(Double) groups significantly reduced CFU compared to the negative control group, with the 12.5 HNT(Double) group displaying the most effective antibiofilm properties among these groups (Figure 3D). This was confirmed in qualitative SEM images, which showed complete depletion of *E. faecalis* biofilm in the 12.5 HNT(Double) group, while the other groups did not show the same efficacy (Figure 3E). A classical study found that 0.5% sodium hypochlorite needed 30 min of contact time to inhibit the growth of *E. faecalis* completely [22,28]. Although the hydrogel did not reach that concentration in our experiment, it was kept in contact with the biofilm on the dentin slice for seven days. This allowed the lower concentration of NaOCl released to work for a longer time and effectively eliminate the contamination.

### 2.7. Chlorine Test

Measuring the release of chlorine from sodium hypochlorite is critical for evaluating its antimicrobial activity, ensuring standardization and safety, and optimizing endodontic treatment protocols. By semi-quantitatively assessing the 12.5 HNT(Double) hydrogel after one, three, and seven days of incubating the samples in PBS containing collagenase, we found a range of 10 mg/L to 20 mg/L, showing burst release after one day, that progressively decreased over time until days three and seven, reaching values of 5 mg/L and 2 mg/L, respectively. The findings agree with previous investigations that demonstrated that a concentration of 5 mg/L did not significantly change DPSC viability after two hours of incubation and reduced it to between 20 and 30% when incubated for twenty-four hours [29]. According to another study, cultured human pulp cells had the highest chance of survival when exposed to the lowest concentration (0.04%) of NaOCl that was tested [21,30]. Our findings demonstrate that the free chlorine levels released by utilizing sodium hypochlorite in the proposed hydrogel did not adversely affect cell viability, as evidenced in Section 2.4. The promising observation paves the way for the development of an innovative treatment protocol aimed at preserving vital pulp cells in revascularization and regeneration scenarios, with speculation that the presence of vital pulp tissue remnants and the Hertwig epithelial root sheath containing mesenchymal stem cells play a crucial role in the success of such cases, following canal disinfection and a reduction in the inflammatory process. In this way, using the proposed hydrogel enables favorable outcomes in regenerative cases while minimizing potential harm to periapical tissues, even in cases where unintentional extrusion extends beyond the periapex.

### 2.8. In Vivo Biocompatibility and Biodegradation

**Figure 4:**
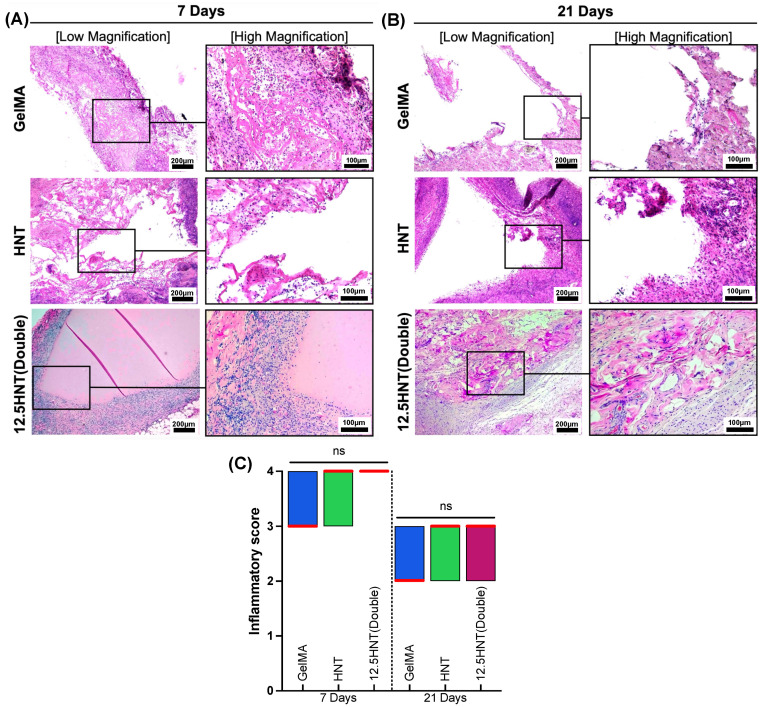
Representative H&E-stained images of GelMA, HNT, and 12.5 HNT(Double) hydrogels implanted in rats’ subcutaneous tissue after 7 days (**A**) and 21 days (**B**). The black square area shows the area elected for high magnification. The analyzed samples’ inflammatory score is displayed through floating bars depicting the minimum to maximum range, with a median line (red) that is prominently marked (**C**). Note a visually slower hydrogel degradation for the 12.5 HNT(Double) compared to the pure GelMA and HNT groups. Moreover, even though HNT and 12.5 HNT(Double) hydrogels evoked higher inflammatory reactions compared to the control (GelMA) at each time point, no statistical differences were found (Kruskal–Wallis/Dunn’s post-hoc, α = 5%, ns: not significant).

In vivo models, which involve studying the behavior and effects of new materials in living organisms, are essential for developing and evaluating new materials in various fields, including biomedicine, pharmaceuticals, and materials science. They can provide crucial information on the material’s interactions with the host tissue, its durability, and its ability to maintain its properties over time. They also allow researchers to better understand the biological response to new materials, including their biocompatibility, toxicity, and overall effectiveness. Based on the prior analysis, GelMA, HNT, and 12.5 HNT(Double) hydrogels were subcutaneously implanted in rats to assess biocompatibility and biodegradation [15]. The representative images (Figure 4) obtained after hematoxylin and eosin (H&E) staining after 7 and 21 days showed that pure GelMA hydrogels led to less inflammation with a median score of 3 compared to the other two groups, which presented mild levels with a score of 4 on day 7, transitioning to minimal inflammation with a score of 2 on day 21, agreeing with previous investigations employing GelMA [31]. Pure HNT hydrogel had a moderate infiltrate, with a median score of 4 on day 7, decreasing to mild, with a median score of 3 on day 14. The 12.5 HNT(Double) hydrogels revealed the same inflammatory cell infiltration pattern at both time points, mainly restricted to the areas close to the hydrogel site but without signs of tissue necrosis. Regarding degradation, all hydrogels showed some degradation after seven days, with the GelMA and HNT groups almost completely reabsorbed after that. Interestingly, the 12.5 HNT(Double) group showed a slower degradation speed, evolving to pronounced resorption after 21 days. Since the major disadvantage of NaOCl in its current clinical form is its cytotoxic effect if injected into the periapical tissues due to the higher concentration, our developed hydrogel has exhibited remarkable biocompatibility, notably by not inducing tissue necrosis. This exciting outcome presents a safer and more viable alternative to traditional NaOCl usage.

## 3. Conclusions

We successfully developed a NaOCl-loaded nanotube-modified GelMA hydrogel that demonstrated cytocompatibility, biocompatibility, biodegradability, and antimicrobial activity against *E. faecalis* as an injectable on-demand drug delivery system that provides sustained release at safe levels of NaOCl for root canal disinfection. Although the present study has made notable advancements, there remains a wealth of knowledge and exploration to pursue regarding utilizing NaOCl-loaded hydrogels. It is crucial to perform rheological analysis and incorporate additional in vivo models that closely resemble endodontic procedures to enhance our understanding further. This will enable a comprehensive comparison of the antimicrobial activity exhibited by the NaOCl hydrogel against other commonly used root canal dressings, such as calcium hydroxide or triple antibiotic paste. By conducting such comparisons, we can gain insights into whether the NaOCl hydrogel has the potential to replace these traditional dressings and offer a distinctive approach to patients undergoing conventional root canal treatments or regenerative endodontic procedures.

## 4. Materials and Methods

### 4.1. Chemical and Materials

The aluminosilicate clay nanotubes (HNT, halloysite, Dragonite HP) were gifted by Applied Minerals Inc. (New York, NY, USA). Sigma-Aldrich (St. Louis, MO, USA) provided the following materials: type-A gel (300 bloom) from porcine skin, methacrylic anhydride (MA), L-glutamine, brain heart infusion (BHI) broth and agar, MQuant^®^ chlorine test strips, as well as sodium hypochlorite (8.25% and 12.5%) aqueous solution. Gibco-Invitrogen (San Diego, CA, USA) supplied Dulbecco’s phosphate-buffered saline (DPBS), alpha-modified Eagle’s medium (α-MEM), fetal bovine serum (FBS), and penicillin–streptomycin. Fisher Scientific (Waltham, MA, USA), TCI America (Portland, OR, USA), Inter-Med, Inc. (Racine, WI, USA), Hoffman-La Roche Ltd. (Basel, Switzerland), and Promega Corporation (Madison, WI, USA) were the respective manufacturers of absolute-200 proof ethanol, lithium phenyl-2,4,6-trimethylbenzoylphosphinate (LAP L0290), ethylenediamine tetra-acetic acid (EDTA), collagenase type I, and CellTiter 96 AQueous One Solution Reagent.

### 4.2. NaOCl-HNT Loading

Halloysite was sieved at 45 μm and used for loading with different concentrations of NaOCl solutions (5.25%, 8.25%, and 12.5% *v*/*v*). To prepare the HNTs, 1.25 g of the sieved halloysite was mixed with 5 mL of the respective NaOCl solution, vortexed for 20 s, and sonicated for 2 h. The mixture was vortexed again for 20 s, placed in a vacuum chamber (Hi-Temp Vacuum, Thermo Scientific Inc., Marietta, USA) overnight, and subjected to an end-to-end mixer for 4 h. Afterward, the vacuum was applied for 1 h, and the mixture was vortexed for 20 s before centrifugation. The HNT + NaOCl solutions were centrifuged twice (3500 rpm, 10 min each), excess liquid was removed, and the samples were stored at 37 °C for seven days until wholly dried. The dried blend was sieved again at 45 μm, and NaOCl-loaded HNTs were obtained and labeled according to the loaded concentration: 5.25%, 8.25%, and 12.5% [15,32].

### 4.3. Gelatin Methacryloyl (GelMA)

The type-A gel (10% *w*/*v*) was dissolved in DPBS and stirred at 300 rpm while being heated to 50 °C. Subsequently, a dropwise addition of 8 mL of MA was performed into the gel solution, followed by stirring the mixture for 2 h to allow the reaction. A volume of 8 mL of DPBS at 40 °C was added to halt the reaction. Dialysis of the mixture was then carried out in DI water, utilizing dialysis tubing with a molecular weight of 12–14 kDa, for one week at a temperature of 45 ± 5 °C, with the water changed every 12 h to remove salts and unreacted monomers. Afterward, the solution was subjected to freezing at a temperature of −80 °C overnight, followed by a seven-day lyophilization process. Subsequently, the lyophilized product was stored at a temperature of −20 °C until it was required for future use [33,34].

### 4.4. Preparation of NaOCl-HNT-GelMA Hydrogels

For GelMA-based hydrogels, 0.4 g of GelMA was carefully dissolved in 4 mL of DPBS at 50 °C while stirring at 300 rpm. Subsequently, 0.3 mg of the photoinitiator LAP was introduced to the solution at the same temperature and stirring conditions. This process yielded the pure GelMA group, consisting of 10% GelMA and serving as the control in the experiment [15,32]. A concentration of 5% *w*/*v* of NaOCl-free and NaOCl-loaded nanotubes with 5.25%, 8.25%, and 12.5% NaOCl concentration were mixed under stirring conditions (300 rpm) for two hours to disperse them into the GelMA solutions to create the respective groups: HNT, 5.25 HNT, 8.25 HNT, and 12.5 HNT. To increase the antibacterial potential, a 10% *w*/*v* concentration of 12.5% NaOCl loaded nanotubes was created—12.5 HNT(Double). To create GelMA and GelMA-modified samples for the different analyses conducted, specific volumes ranging from 100 to 150 μL of the prepared solutions were carefully placed into silicone molds specifically designed for this purpose and photo-crosslinked for 30 s on both sides using a curing LED light (Bluephase, Ivoclar Vivadent, Amherst, NY, USA) with a broadband spectrum of 385−515 nm [15,32].

### 4.5. Chemo-Morphological Characterization

To investigate the pore morphology of GelMA and nanotube-modified GelMA hydrogels, cylindrical-shaped samples measuring 6 mm in diameter and 10 mm in thickness were prepared following the previously described method. These samples were subjected to analysis using scanning electron microscopy (SEM) with a Tescan MIRA3 FEG-SEM instrument from Tescan USA Inc. (Warrendale, PA, USA). Prior to SEM imaging, the samples (*n* = 5/group) underwent freeze–drying and were cross-sectioned and sputter-coated with Au-Pd. The elemental composition of the samples was assessed through elemental mapping using energy-dispersive spectroscopy (EDS), and backscattered electron diffraction (EBSD) was employed with an EDAX Hikari EBSD camera attached to the scanning electron microscope [15].

### 4.6. Mechanical Characterization

To evaluate the compressive modulus, cylindrical-shaped samples with dimensions of 8 mm diameter × 3 mm thickness were prepared. The samples (*n* = 5/group) underwent a 24 h incubation period in DPBS at a temperature of 37 °C, followed by careful blot-drying to remove any excess liquid. Unconfined compression tests were performed at room temperature using a strain rate of 2 mm/min. The tests were conducted using an ADMET Inc. expert 5601 machine (Norwood, MA, USA). The compressive modulus was determined by analyzing the slope of the stress–strain curves within the linear region corresponding to a strain range of 5–10% [35].

### 4.7. Swelling and Enzymatic Degradation

The degree of hydration was measured to evaluate the swelling capacity of GelMA-based hydrogels. Wet samples, with dimensions of 6 mm in diameter and 2 mm in thickness (*n* = 5/group), were immersed in DPBS at 37 °C for 24 h. Subsequently, the samples were carefully dried by blotting them with low-lint-content tissue paper. The wet weights (Ww) were measured using an analytical balance, and the samples were subsequently lyophilized to obtain dry weights (Wd). The swelling rate (%) was calculated as (Ww − Wd)/Wd × 100 [15,36].

To study the degradation of hydrogels, we placed five cylindrical samples per group into glass vials (VWR International, LLC in Rander, PA, USA) filled with 5 mL of DPBS and 1 U/mL of collagenase type I (MMP1). The vials were then incubated at a temperature of 37 °C. Every sample was taken out of the solution at specific intervals, ranging up to 7 days. After that, it was dried using blotting and weighed with an analytical balance. The collagenase-enriched solutions were replaced every three days to ensure constant enzyme activity. The degradation ratio of the hydrogel was calculated using the following equation:

Degradation ratio (%) = Wt/Wo × 100, where Wt is the residual wet weight at different time points, and Wo is the initial wet weight [15,36].

### 4.8. Cytotoxicity

To investigate the potential cytotoxicity of NaOCl-loaded nanotube-modified GelMA, cylindrical samples with dimensions of 6 mm in diameter and 2 mm in thickness were prepared for an in vitro assay following the guidelines provided by the International Standards Organization (ISO10993-5: tests for cytotoxicity—in vitro methods) [37]. The samples were subjected to UV treatment on both sides for 30 min to ensure disinfection. Each sample was then individually placed in a sterile glass vial containing 5 mL of α-MEM supplemented with 10% FBS, L-glutamine, 1% penicillin–streptomycin, and 1 U/mL of collagenase-type I. The vials with the samples were incubated at a temperature of 37 °C for a maximum period of 7 days. Aliquots of 500 μL were collected from each vial at predetermined time points to assess the cytotoxicity. An equal amount of the collected aliquots was added back to each vial to maintain a constant extraction volume. Prior to exposing the cells, the collected aliquots were filtered through a 0.22 μm membrane [38].

This study utilized stem cells from human exfoliated deciduous teeth (SHEDs), generously provided by Dr. Jacques Nör from the University of Michigan, School of Dentistry. The SHEDs were cultured in α-MEM supplemented with 10% FBS, 1% L-glutamine, and 1% penicillin–streptomycin. The cell cultures were maintained in a 5% CO_2_ incubator at a temperature of 37 °C. Cells between passages 4 to 7 were used. For cell adhesion, SHEDs were seeded at a 2.5 × 10^3^ cells/well density in 96-well plates. Following a 24 h incubation period, the collected extracts (100 μL) derived from the hydrogels were added to the cultured cells and incubated for an additional 24 h. Subsequently, CellTiter 96 AQueous One Solution Reagent (20 μL) was introduced to the wells, and the resulting dye was allowed to react for 2 h at a temperature of 37 °C in a humidified atmosphere containing 5% CO_2_. Absorbance values were measured at 490 nm against a blank column using a SpectraMax iD3 device (Molecular Devices, San Jose, CA, USA). As a positive control, SHEDs cultured in complete α-MEM were employed. The obtained absorbance values were converted to percentages and compared with those acquired from the respective test groups [15].

### 4.9. Antibacterial Properties

The agar diffusion assay of *E. faecalis* (ATCC 19433) was carried out to determine the antimicrobial properties of NaOCl-loaded nanotubes (direct contact) and NaOCl-loaded nanotube-modified GelMA hydrogels (indirect contact). To assess the indirect antimicrobial effects of MMP-responsive hydrogels, cylindrical samples with dimensions of 6 mm in diameter and 2 mm in thickness were prepared (*n* = 4/group). Prior to the experiment, the samples were subjected to 30 min of UV treatment on both sides to ensure disinfection. Each sample was then individually placed in glass vials containing 5 mL of sterile PBS supplemented with 1 U/mL of collagenase type I. The vials were incubated at a temperature of 37 °C. At predetermined intervals, which spanned up to 7 days, 500 μL aliquots were withdrawn from each vial. An equal amount of the same solution was replenished after each withdrawal to maintain a consistent extraction volume. The collected aliquots were subsequently stored at a temperature of −20 °C for further use in succeeding analyses. For the direct contact, 60 mg of NaOCl-loaded nanotubes (*n* = 4/group) was pressed using a 1-ton Arbor press with a metal mold of 6 mm diameter. After the samples were prepared, they were disinfected as described before and kept at room temperature until further use. Direct contact with hydrogel was also tested. Cylindrical samples with dimensions of 6 mm in diameter and 2 mm in thickness were prepared (*n* = 4/group) and subjected to UV as described before. The microorganisms were cultured in 5 mL of BHI broth for 24 h. The resulting bacterial suspension was adjusted using spectrophotometry to obtain a concentration of 3 × 10^8^ CFU/mL and then swabbed (100 μL) onto BHI agar plates to create a bacterial lawn [15]. Each bacterial plate was divided into four zones, namely a zone treated with 20 μL of 2% chlorhexidine digluconate (CHX; positive control) or 20 μL of the GelMA-based aliquots or the pressed nanotubes (3 zones/plate randomly assigned). After 24 h of incubation, the apparent growth inhibition zones’ diameters (in mm) were measured [15].

### 4.10. Antibiofilm Properties

For the antibiofilm analysis, we excluded the group 5.25 HNT since the hydrogels did not demonstrate a favorable antimicrobial activity in time-course assays. To carry out the experiment, intact molar human teeth extracted from patients without caries were obtained from the Community Dental Center at the University of Michigan in Ann Arbor, MI, USA. The collected teeth were thoroughly cleaned and stored in a 0.01% sodium azide solution to preserve their integrity. Subsequently, the crowns of the teeth were carefully sectioned using an IsoMet 1000 Precision Saw (Buehler, IL, USA), resulting in dentin slices with a thickness of 2 mm. After polishing the dentin slices with SiC papers of 600 grit, a series of treatments was conducted. The dentin slices were first exposed to 2.5% NaOCl solution for 3 min, followed by treatment with 17% EDTA solution for 3 min in an ultrasonic bath. Subsequently, the slices were rinsed with saline for 10 min and then subjected to autoclaving at 121 ℃ for 20 min for sterilization [27]. *E. faecalis* (ATCC 19433) was cultured in BHI broth and adjusted to a cell density of approximately 7.5 × 10^7^ CFU/mL. Dentin slices were placed in 24-well plates with BHI broth and the bacterial suspension and incubated for seven days with regular broth changes to support biofilm growth. The infected dentin slices (*n* = 6/group) were divided into six groups: GelMA, HNT, 8.25 HNT, 12.5 HNT, 12.5 HNT(Double), and an untreated 7-day-old biofilm (negative control). After 7 days, unattached bacteria were rinsed off with PBS. Each hydrogel formulation (50 μL) was applied to the biofilm/dentin samples and photo-crosslinked for 30 s. The samples were incubated in sterile PBS with collagenase type I for seven days. CFU/mL determination (*n* = 4) and SEM imaging (*n* = 2) were performed [27]. For CFU/mL assessment, the samples were transferred to centrifuge tubes with 1000 μL of saline for serial dilution, plated on BHI agar plates, and incubated at 37 °C for 24 h to calculate CFU/mL. The samples were rinsed with PBS and fixed overnight in 2.5% glutaraldehyde for SEM analysis. After fixation, the samples underwent dehydration using a series of alcohol solutions with increasing concentrations. Subsequently, the samples were treated with a hexamethyldisilazane solution. Finally, before SEM imaging, the dentin slices were coated with Au−Pd.

### 4.11. Chlorine Test

After analyzing the findings from our previous methods, we selected the 12.5 HNT(Double) hydrogel for a chlorine release test due to its effective antibacterial and antibiofilm properties. To confirm chlorine release, we performed a semi-quantitative exploratory test using MQuant^®^ chlorine test strips using the same aliquots collected for the indirect antimicrobial test. These strips react with chlorine, producing a violet dye that can be compared to a color scale to confirm the reaction zone visually.

### 4.12. In Vivo Biocompatibility and Biodegradation

The animal research conducted in this study adhered to the ARRIVE guidelines and followed the protocols of the local Institutional Animal Care and Use Committee (PRO00010329). A sample size calculated from previous studies of eight male Fischer 344 (Envigo RMS, Oxford, MI, USA) rats (6 weeks old, weighing 300–320 g) were selected for the experiments. The surgical procedures were performed on rats under general anesthesia induced by intraperitoneal administration of ketamine (50 mg/kg, Hospira, Inc., Lake Forest, IL, USA) and xylazine (5 mg/kg, Akorn, Inc., Lake Forest, IL, USA). Following thorough antibacterial and antibiofilm analyses, it was determined that solely the 12.5 HTN(Double) hydrogel demonstrated adequate bacterial reduction properties. Consequently, this was the only tested group evaluated in vivo, in addition to controls. Subcutaneous pockets were made through short dorsal skin incisions (10 mm length), and GelMA, HNT, and 12.5 HTN(Double) hydrogels (cylindrical samples, 8 mm diameter × 3 mm thick) were implanted (*n* = 4/group). The surgical wounds were closed with Coated Vicryl^®^ polyglactin 910 sutures (Ethicon Endo-Surgery, Inc., Cincinnati, OH, USA). The animals were euthanized with CO_2_ inhalation after 7 or 21 days. The hydrogels that were implanted, along with the surrounding peri-implantation tissue, were retrieved. The samples were fixed overnight in 10% buffered formalin, embedded in paraffin, sectioned into 6 μm-thick slices, and stained with H&E to assess the presence of inflammatory cells while evaluating hydrogel degradation. A single calibrated operator, blinded to the experimental conditions, used light microscopy (Echo Revolve, BICO Company, San Diego, CA, USA) to investigate the tissue reactions close to the hydrogels’ implantation sites. The tissue reactions were scored using a scale adapted from previous studies: 1 (few inflammatory cells or no reaction), 2 (mild reaction with less than 25 cells), 3 (moderate reaction with 25 to 125 inflammatory cells), and 4 (severe reaction with 125 or more inflammatory cells) [39,40].

### 4.13. Statistical Analysis

The statistical analyses in this study were conducted using GraphPad Prism software (Version 9.5.1). We used one-way or two-way analysis of variance (ANOVA) with Tukey’s and Sidak’s post-hoc test to determine statistical significance, with a significance level of *p* < 0.05. For analyzing inflammatory scores, we used Kruskal–Wallis with Dunn’s post-hoc test.

## Data Availability

All data and materials are available on request from the corresponding author. The data are not publicly available due to ongoing research using a part of the data.
